# Osteophyte formation causes neurological symptoms after anterior cervical discectomy and fusion (ACDF): A case report

**DOI:** 10.3389/fsurg.2022.1029743

**Published:** 2023-01-13

**Authors:** Haiming Jin, Jiangtao Luo, Yuhan Jiang, Jinghao Lin, Junchen Jiang, Rufeng Ren, Weiyuan Fang, Yaosen Wu, Xiangyang Wang

**Affiliations:** ^1^ Division of Spine Surgery, Department of Orthopaedics, The Second Affiliated Hospital and Yuying Children's Hospital of Wenzhou Medical University, Wenzhou, China; ^2^ Key Laboratory of Orthopaedics of Zhejiang Province, The Second Affiliated Hospital and Yuying Children's Hospital of Wenzhou Medical University, Wenzhou, China; ^3^ School of the Second Clinical Medical Sciences, Wenzhou Medical University, Wenzhou, China

**Keywords:** osteophyte, ACDF = anterior cervical discectomy and fusion, myelopathy, cervical open-door laminoplasty, strong fixation

## Abstract

Spinal surgeons have been drawn to the incidence of osteophytes following intervertebral disc degeneration in clinical practice. However, the production of osteophytes, particularly in the spinal canal, after anterior cervical discectomy and fusion (ACDF) is uncommon. We described a 42-year-old male patient who underwent C4–6 ACDF due to cervical stenosis two years prior in another public hospital in the province. His primary symptoms were significantly relieved, but he developed new pain and weakness in his right leg six months after surgery. The imaging results revealed a large posterior osteophyte at C5/6, compressing the spinal cord anteriorly. Accordingly, we performed cervical open-door laminoplasty to decompress the spinal cord. The patient's clinical symptoms had significantly improved at the one-year follow-up. This case seeks to inform surgeons that cautious, routine follow-ups are necessary for the event that a severe intracanal osteophyte develops at the operated level following ACDF. The comprehensive osteophyte removal and strong fixation at the operative level during ACDF warrant more consideration as these procedures may lower the incidence of new osteophytes. Additionally, surgical procedures may be required.

## Introduction

An osteophyte is a fibrocartilage-capped bony protrusion that is a characteristic of osteoarthritis; it is unusual for vertebral osteophytes to form following disc degeneration (1, 2). Theoretically, the fusion of the vertebrae in the spine will increase stress loads and strains at the adjacent segments, which could result in the development of osteophytes in the segments adjacent to the fused vertebrae (3). Anterior cervical discectomy and fusion (ACDF) is a routinely performed spinal fusion procedure for decompressing the cervical cord (4), and having undergone this procedure is a potential risk factor for developing osteophytes in the adjacent segments (5). However, in our assessment of the English scientific literature, we found that the production of symptomatic osteophytes at the operative level is highly unusual after ACDF as only two cases of osteophytes in the posterior region of the operated disc were described (6, 7). In the current work, we present a unique case of a large osteophyte that developed after ACDF. Osteophyte growth within the spinal canal generated clinical symptoms of neural compression. A posterior one-sided open-door laminoplasty was performed to decompress the spinal cord to relieve the patient's myelopathic symptoms.

## Case report

**Figure 1:**
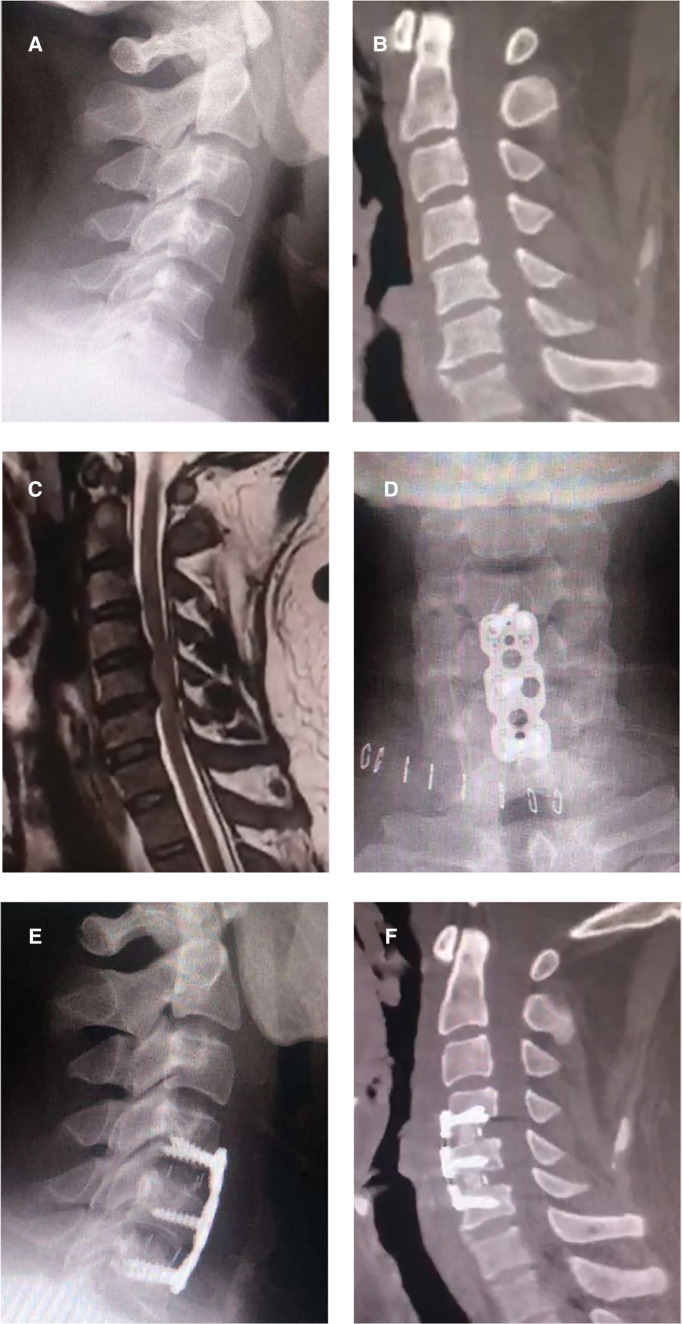
Preoperative and postoperative images of anterior cervical discectomy and fusion. (**A**) A preoperative lateral x-ray image of the patient; (**B**) A preoperative sagittal CT image of the patient; (**C**) A preoperative sagittal MR image, revealing spinal stenosis at the C4/5 and C5/6 levels secondary to central disc herniation; (**D–F**) Postoperative x-ray and CT images, revealing accurate placement of the plates, screws, and cages.

A 42-year-old male patient was diagnosed with cervical stenosis three years ago, with limb numbness for two weeks and aggravation for one week. Imaging results demonstrated spinal stenosis at the C4/5 and C5/6 levels secondary to central disc herniation (Figures 1A–C). He received treatment at the time in another public hospital in the province with standard ACDF at both levels (C4/5, C5/6) utilizing the right-sided approach (Figures 1D–F). A steel plate was screwed to the C4 and C6 vertebral bodies, with both two screws attached to the C4 and C6 and another screw attached to the C5. The patient experienced significant improvement from his initial symptoms following surgery, but he developed new pain and weakness in his right leg six months later. Symptoms persisted despite a 2-year course of nonsurgical treatments that included physical therapy, nonsteroidal anti-inflammatory, and neurotrophic medication. The symptoms worsened one month before admission to our hospital, and the patient developed new symptoms of limb numbness and weakness accompanied by unstable walking, limiting his ability to perform regular tasks and ambulate. Physical examination revealed decreased sensations in both the upper and lower limbs, whereas the symptoms were more severe on the right side than on the left. His muscle strength was grade 4 for the upper limbs, grade 3 for the left leg, and grade 2 for the right leg. Hoffman's and Babinski's signs were positive on both sides.

**Figure 2:**
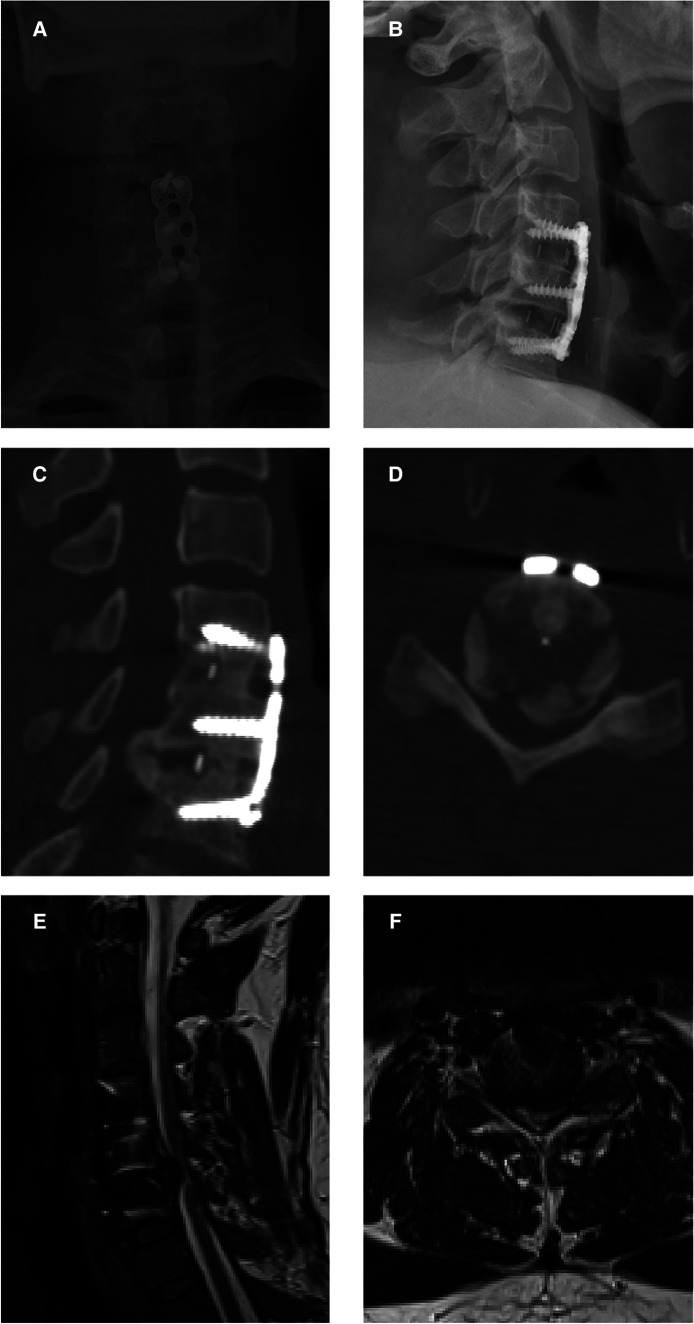
Preoperative images before open-door laminoplasty. (**A,B**) Preoperative x-ray images showing a large posterior cervical osteophyte at the level of C5/6. (**C**) A preoperative sagittal CT image showing a large heterotopic bone achieved from the posterior side of the C4 inferior end plate to the posterior side of the C5 superior end plate. (**D**) A transverse CT image revealing evident cervical stenosis and anterior spinal cord compression due to ossification. (**E,F**) Preoperative MR images showing the narrow space of the spinal canal and a T2-weighted hyperintense intramedullary signal at the C5/6 level.

The x-ray images revealed a large posterior cervical osteophyte at the level of C5/6 (Figures 2A,B), which did not exist at the time of the initial procedure. The C4–6 Cobb angle was 12.8°. The computed tomography (CT) scan provided more information about the osteophyte. The preoperative sagittal CT images revealed a large heterotopic bone that stretched from the posterior side of the C5 inferior end plate to the posterior side of the C6 superior end plate (Figure 2C). It also demonstrated the segmental fusion status following ACDF, revealing bridging trabeculae at the C4/C5 level, which was considered fused, and a bony gap at the C5/C6 level, which was termed fused poor. Transversal CT imaging revealed evident cervical stenosis, and ossification compressed the spinal cord anteriorly, resulting in a narrow space laterally for the spinal cord, dura mater, and cerebrospinal fluid (Figure 2D). The preoperative MR images also revealed a narrow spinal canal space and a T2-weighted hyperintense intramedullary signal at the C5/6 level (Figures 2E,F).

The symptoms failed to improve with nonoperative management; as such, the patient was advised to undergo surgical decompression and posterior stabilization *via* laminoplasty under general anesthesia. We performed cervical open-door laminoplasty at C3–7 to widen the space of the cervical vertebral canal, particularly at the C5/6 level, to assure the postoperative effect of decompression surgery. A cervical midline skin incision was made from C2 spinous processes to C7. The C3–C7 laminae were exposed following the installation of the automatic retractor. Open-door laminoplasty was performed by constructing bilateral gutters at the intersection of the laminae and the medial aspect of the lateral mass. The left side was opened, while the right-side gutter served as a hinge. The laminae from C3 to C7 were fully opened and held in place with mini plates placed between the laminae and lateral mass. The wound was then extensively irrigated and closed in layers after homeostasis was achieved. The postoperative course went smoothly, and the patient felt considerably better and presented no symptoms.

**Figure 3:**
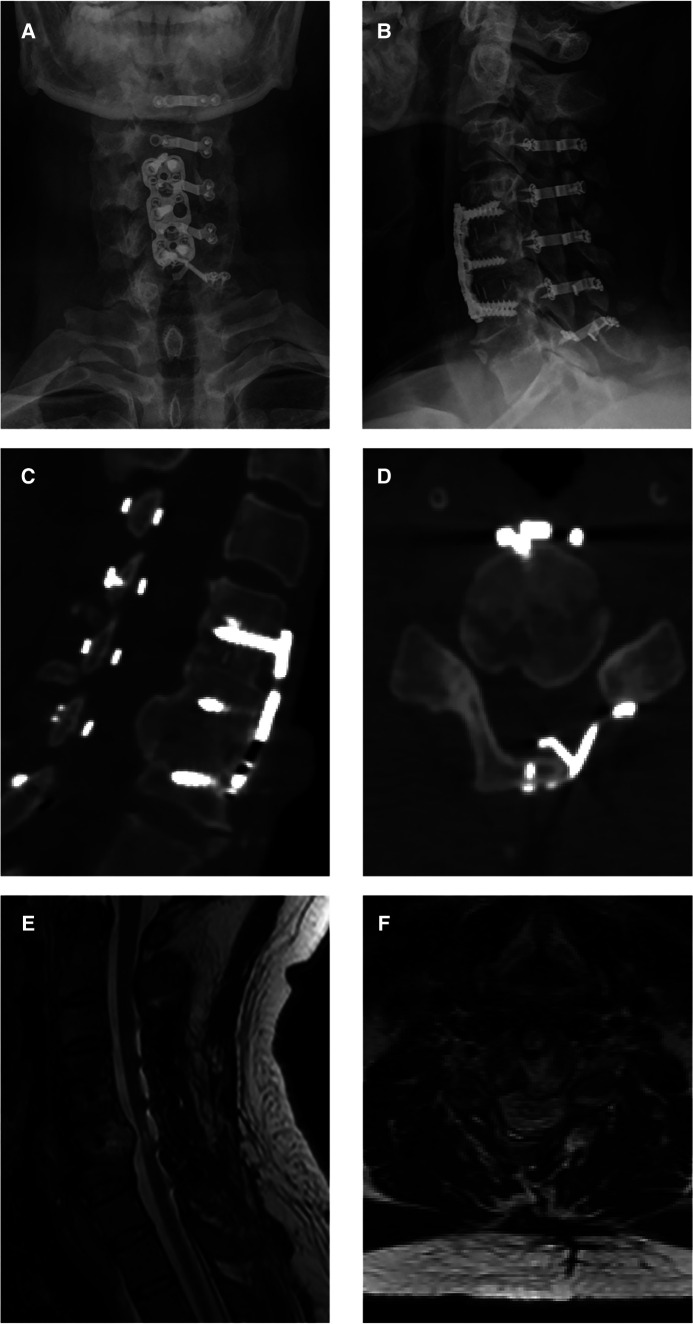
One-year follow-up images after open-door laminoplasty. (**A,B**) Postoperative x-ray images showing the accurate positions into which the plates and screws were installed. (**C,D**) Postoperative CT images showing direct adherence of all plates to the host bone, creating a new, wide canal space. (**E,F**) Postoperative MRI images showing decompression of the spinal cord at the C5/6 level.

The x-ray and CT scans during the one-year follow-up revealed that all the plates had adhered directly to the host bone, widening the canal space (Figures 3A–D). The MRI scan at the one-year follow-up showed decompression at the C4/5 level (Figures 3E,F). The muscle strength grades of the left upper limb, right upper limb, left lower limb, and right lower limb improved to 5, 5, 4, and 3, respectively, after the operation.

## Discussion

A vertebral osteophyte is a typical osteoarthritic characteristic that is defined as an aberrant bony growth or bone spur that occurs along intervertebral joints (8). Emerging evidence indicates that age, acute injury, endplate sclerosis, and intervertebral disc degeneration are potential risk factors for the formation of vertebral osteophytes (8, 9). The fusion of spine segments may also result in osteophyte formation at adjacent segments (3). In contrast, no osteophytes were found at the adjacent segments in our case, and a severe intracanal osteophyte grew at the operative level without the application of bone morphogenetic proteins. The intracanal osteophyte triggered severe neurological symptoms. This is, to the best of our knowledge, the first time such a case has been reported.

The precise cause of osteophytes appears intricate and elusive. In successful fusion cases of cervical surgery, the vertebral range of motion (ROM) at the operative level is severely limited. At the same time, any persisting ROM and an abnormal instantaneous axis of rotation (IAR) at the operative level may trigger osteophyte formation in cases of cervical instability by loosening the instrumentation used for internal fixation. Osteophyte formation may be a self-regulatory mechanism as it increases the contact surface and restricts excessive motion. In this view, applying appropriate internal fixation devices such as an anterior plate and screws, as well as appropriate postoperative management, can help to avoid screw loosening and enhance the spine stability at the operative level. In this case, we strongly believe the first operation caused cervical instability. The surgeon managed to secure one screw fixation at C4 and C5 levels during the first surgery, however, the second screw at the C4 level violated the endplate. Poor C4 and C5 fixations generated a lever arm on the solid C6 fixation. The lever arm resulted in a mobile segment with unusual ROM and an abnormal IAR, which could explain the formation of an osteophyte. The inconspicuous remnant osteophyte following ACDF could be another major cause of osteophyte in the present case. We noted that osteophyte formation was primarily on the left side, which corresponded to the location of the marginal remnant posterior osteophyte detected in postoperative CT images following ACDF. Progenitor cells derived from muscle and bone are thought to potentially differentiate into osteoprogenitor cells, supplying osteoblast precursors in the formation of new bone (10, 11). The manipulation of bone and muscle during surgery may allow progenitor cells to spread into nearby well-vascularized soft tissue. Choosing the appropriate operative technique, honing one's operative skills, and keeping the surgical field free might, therefore, assist lowering the rate of osteophytes. In addition, post-surgery inflammatory responses may be a factor in the progression of osteophyte formation (12). Studies have demonstrated that NSAIDs significantly lower the incidence of heterotopic ossification after cervical arthroplasty (13). This “heterotopic ossification” after cervical arthroplasty does not occur within soft tissue, and the mechanism is presumed to be related to the aggravation of preoperative osteophytes with aseptic inflammatory hyperplasia and dynamic loading stimulation (14, 15), which is highly comparable to the formation of vertebral osteophyte in the present case. Bone fusion and heterotopic ossification are both regarded as indicators of individual osteogenic capacity in patients (16), and we speculate that accessible NSAIDs may inhibit osteophyte formation through a similar mechanism. Furthermore, low-dose NSAIDs are frequently used for postoperative analgesia and have been shown not to influence the fusion rate after the lumbar fusion procedure (17). It is critical to note that an infection, postoperative hematoma, or persistent abnormal motion at the surgical site may aggravate inflammation. Patients with potential infections should take NSAIDs to manage them. Moreover, treatment with bisphosphonate and alendronate could slow osteophyte progression (18, 19). The recombinant human bone morphogenetic protein-2 (rhBMP-2) has been identified as a potential accelerating factor for osteophyte formation 6, while the application of barriers to prevent BMP exposure to soft tissue and neural elements may minimize its effects (20).

**Figure 4:**
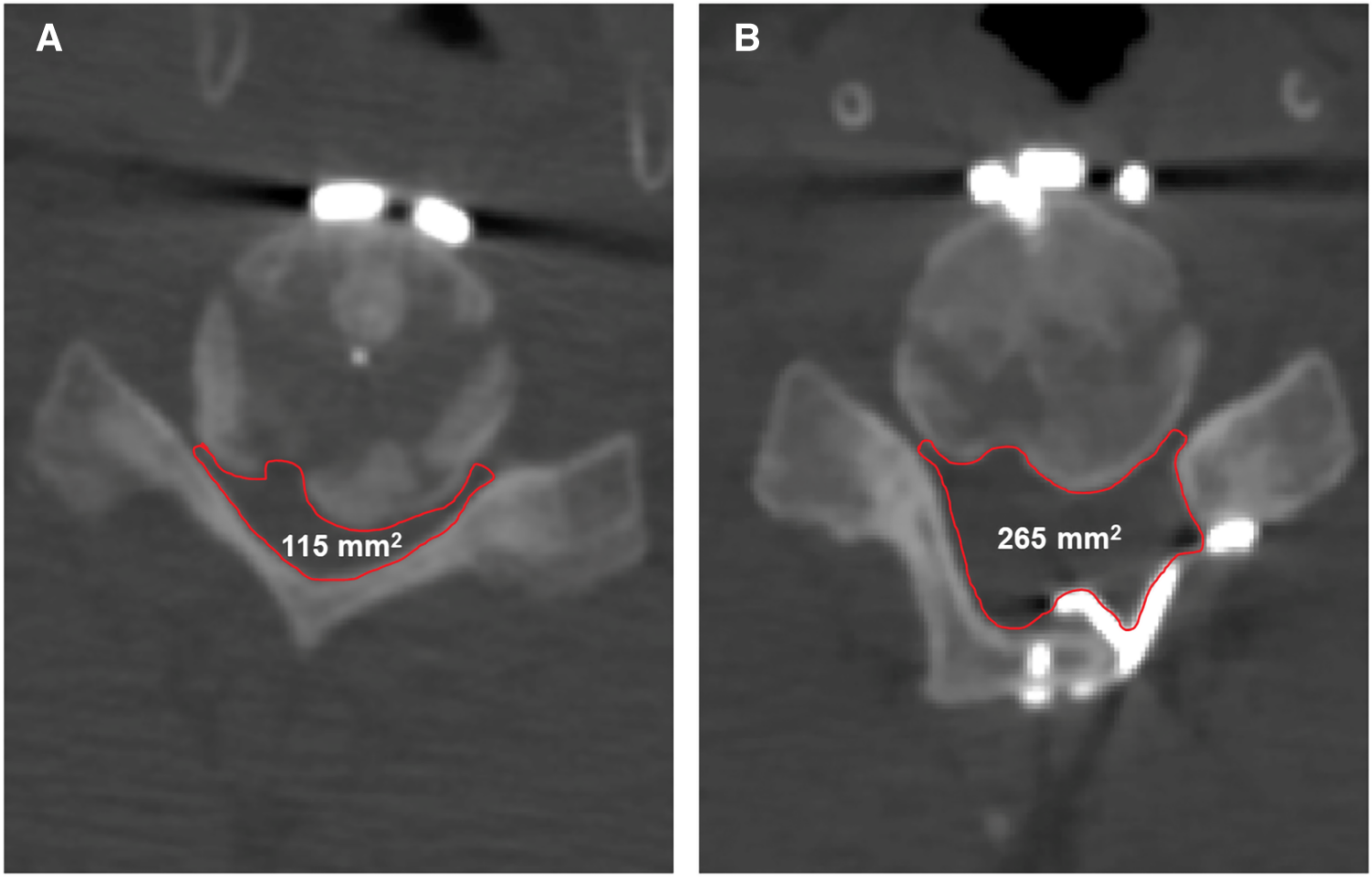
The changes in the space in the spinal canal from preoperative to postoperative. (**A**) The preoperative space in the spinal canal at the C5/6 level is 115 mm^2^. (**B**) The postoperative space of the spinal canal at the C5/6 level is 265 mm^2^.

The present work observed bone development at the posterior region of the C5/6 level (Figure 2). While research into the fate of the posterior osteophyte following anterior cervical fusion surgery is scarce and contradictory, recent studies have shown that spontaneous diminution of the posterior osteophyte is extremely unusual. As a result, suitable surgical procedures are useful in improving the prognosis. All surgical attempts to resect this heterotopic bone run the risk of further spinal cord compression and may cause cerebrospinal leakage owing to tight adhesion or ossification of the dura. Moreover, dissecting scar adhesion between the plate and surrounding tissues to remove the fixation devices may result in several complications. This is why we first performed posterior decompression; which saw a gradual improvement in the patient's symptoms after surgery. Postoperative CT scan revealed that the space in the spinal canal expanded from 115 mm^2^ to 265 mm^2^ at the C5/6 level (Figure 4), whereas the postoperative MRI scan showed complete decompression of the spinal cord (Figures 3E,F). Given the patient's clinical improvement, we recommended that he receive rehabilitation therapy and proceed with follow-ups to detect any progression of osteophytes or the emergence of new symptoms.

## Conclusion

Patients who receive ACDF occasionally develop osteophytes. The massive intracanal heterotopic bone and poor clinical symptoms found in the present case distinguish it from others. Nonetheless, we performed a successful surgical posterior decompression by open-door laminoplasty. The case report aims to raise awareness among surgeons that a severe intracanal osteophyte may develop after ACDF, and that cautious, regular follow-ups are warranted. Furthermore, to reduce the rate of postoperative osteophytes, surgeons should focus more on the comprehensive removal of osteophytes, strong fixation at the operative level, and a clear surgical field during ACDF. Surgical interventions may also be required.

## Data Availability

The raw data supporting the conclusions of this article will be made available by the authors, without undue reservation.
